# Subtype-Specific Macular Microvascular Changes in Accommodative Esotropia Assessed by Optical Coherence Tomography Angiography

**DOI:** 10.1167/iovs.67.10.68

**Published:** 2026-08-27

**Authors:** Soner Güven, Mutluay Bozoklu, Sedat Arıkan

**Affiliations:** 1 Kayseri City Training and Research Hospital, Department of Ophthalmology, Kayseri, Turkey

**Keywords:** accommodative esotropia (AE), optical coherence tomography angiography (OCTA), microvasculature, choriocapillaris (CC), pediatric strabismus

## Abstract

**Purpose:**

The purpose of this study was to evaluate macular microvascular alterations in children with accommodative esotropia (AE) using optical coherence tomography angiography (OCTA) and to determine whether these changes differ across AE subtypes independent of refractive and biometric confounders.

**Methods:**

This prospective cross-sectional study included 34 children with AE (68 eyes) and 35 age-matched healthy controls (70 eyes). AE was classified into refractive (*n* = 8), high accommodative convergence to accommodation (AC/A) ratio (*n* = 10), and partially accommodative (*n* = 16) subtypes. All participants underwent comprehensive ophthalmic examination and OCTA imaging. Vessel density (VD) was measured in the superficial capillary plexus (SCP), deep capillary plexus (DCP), choriocapillaris (CC), and outer retina (OR) across five macular regions. Generalized estimating equation models adjusted for spherical equivalent and axial length (AL) were used for comparisons.

**Results:**

The AE and control groups were age-matched (9.2 ± 2.3 vs. 9.8 ± 2.8 years, *P* = 0.356). No significant differences were found in SCP between groups. CC analysis revealed significantly reduced VD confined to the superior quadrant in high AC/A ratio (*P* = 0.005, q = 0.03) and partially accommodative (*P* < 0.0001, q < 0.001) AE subtypes compared with controls. OR analysis showed superior quadrant reductions in high AC/A ratio AE (*P* = 0.010, q = 0.04). DCP reductions in partially accommodative AE (*P* = 0.027, q = 0.09) and CC reductions in refractive AE (*P* = 0.041, q = 0.12) did not survive false discovery rate (FDR) correction. All other quadrants showed no significant differences.

**Conclusions:**

Children with AE exhibit significant, layer-specific microvascular alterations predominantly confined to the superior quadrant, with distinct patterns across AE subtypes. After FDR correction, CC findings in high AC/A ratio and partially accommodative subtypes, and OR findings in high AC/A ratio subtypes, remained significant. These findings suggest that chronic accommodative effort and convergence stimulation may influence retinal microvasculature, providing new insights into the pathophysiology of AE.

Accommodative esotropia (AE) is a common form of concomitant strabismus characterized by inward deviation of the visual axes, typically resulting from uncorrected hypermetropia or an abnormal accommodative convergence to accommodation (AC/A) ratio.^1^ AE is classified into three subtypes: refractive (fully correctable with hyperopic correction), partially accommodative (residual esotropia despite full correction), and high AC/A ratio (convergence excess type).^2^ Whereas optical and surgical management strategies are well-established, the potential morphological and microvascular alterations accompanying chronic accommodative effort and convergence stimulation remain largely unexplored.^3^

Recent advances in ocular imaging have revealed that strabismus may be associated with structural changes beyond the extraocular muscles.^4^ Children with AE exhibit shallower anterior chambers and limited forward lens movement during accommodation compared with hyperopic controls.^4^ Longitudinal studies demonstrate that ocular biometric components develop at different rates in infantile versus late-onset AE, with slower axial elongation in the late-onset group.^5^ Optic nerve head morphology is also affected, with esotropic children showing significantly smaller cup volume and cup-to-disc ratio independent of refractive error and axial length (AL).^6^ Most notably, patients with concomitant esotropia exhibit increased thickness of nasal intraretinal layers, particularly the ganglion cell and inner nuclear layers, suggesting that strabismus involves retinal structural adaptations.^7^

Optical coherence tomography angiography (OCTA) enables noninvasive, reproducible quantification of retinal microvasculature across four layers: superficial capillary plexus (SCP), deep capillary plexus (DCP), outer retina (OR), and choriocapillaris (CC).^8^ In healthy eyes, retinal perfusion correlates with thickness, supporting a structure-perfusion relationship that may be relevant to understanding strabismus-related adaptations.^9^ In amblyopic populations, OCTA has revealed reduced vessel density (VD) in the SCP and DCP, with negative correlations between visual acuity and VD.^10^ Investigations in exotropic strabismus suggest that the consistently deviating eye may exhibit region-specific microvascular reductions.^11^ However, despite growing evidence of structural ocular changes in AE and the established utility of OCTA in detecting subclinical vascular alterations, no published study has specifically investigated macular microvascular changes in children with AE.^4^^–^^6^

The distinct pathophysiology of AE—characterized by sustained accommodative effort and associated convergence stimulation, rather than constant ocular deviation alone—warrants independent investigation. Understanding whether these chronic processes induce measurable microvascular alterations, and whether such changes differ across AE subtypes, could provide novel insights into disease mechanisms, and potentially identify new biomarkers for disease severity or treatment response.

The present study aims to address this knowledge gap by evaluating macular microvascular alterations in children with AE using OCTA, and to determine whether these changes are independent of refractive and biometric confounders. A secondary aim was to explore whether microvascular characteristics differ among refractive, high AC/A ratio, and partially accommodative AE subtypes compared with control eyes.

## Methods

### Study Design and Ethical Approval

This prospective, cross-sectional study was conducted at Kayseri City Training and Research Hospital, Turkey, between March 2025 and January 2026. Ethical approval was obtained from the University of Health Sciences Kayseri City Hospital Institutional Review Board (Protocol #382), and the study adhered to the Declaration of Helsinki. Written informed consent was obtained from all parents or legal guardians.

### Participants

The study included children aged 5 to <16 years diagnosed with AE and age-matched healthy controls. AE was defined as esotropia that resolves or significantly decreases with full hypermetropic correction, and was classified into three subtypes: refractive (normal AC/A ratio, aligned with full correction), partially accommodative (residual esotropia >10 prism diopters [PD] after full correction), and high AC/A ratio (AC/A ratio >5:1 or near esotropia ≥10 PD greater than distance).^2^

Inclusion criteria for patients with AE were: (1) best-corrected visual acuity (BCVA) 20/20 or better in each eye; (2) hyperopia +2.00 to +6.00 diopters (D) spherical equivalent (SE) with cylinder <2.00 D; (3) age 5 to <16 years; (4) no amblyopia (interocular difference <2 lines); and (5) ability to cooperate with OCTA imaging. None of the included patients with AE had a history of amblyopia treatment (including patching or atropine penalization). All patients achieved BCVA of 20/20 or better in each eye without prior amblyopia therapy.

Control inclusion criteria were: (1) BCVA 20/20 or better; (2) refraction ± 1.00 D with cylinder <1.00 D; (3) orthophoria or small heterophoria (<5 PD); and (4) no strabismus or ocular pathology.

Exclusion criteria for both groups included nystagmus, amblyopia, paralytic/restrictive strabismus; prior ocular surgery; prematurity (<36 weeks); organic eye disease; neurological or systemic conditions affecting vision; use of topical/systemic medications affecting ocular structures; anisometropia >1.50 D; and poor-quality OCTA scans (quality index <40 or significant artifacts).

### Sample Size Calculation

Sample size was calculated using G*Power. Based on an expected effect size of d = 0.80 from previous OCTA studies in strabismus, a 2-tailed independent *t*-test with α = 0.05 and power = 0.80 required 52 participants (26 per group).^11^ To account for exclusions, we aimed to recruit 70 participants (35 patients and 35 controls). This power calculation was performed strictly for the primary comparison (AE overall versus controls) and not for the secondary exploratory subgroup analyses (refractive, high AC/A ratio, and partially accommodative AE subtypes versus controls). Accordingly, these subgroup comparisons were not powered for definitive inference, and the findings should be interpreted as hypothesis-generating.

### Ophthalmic Examination

All participants underwent comprehensive evaluation including BCVA using age-appropriate logMAR charts; cycloplegic refraction with cyclopentolate 1%; AL measurement using IOL Master 700 (Carl Zeiss Meditec); and anterior/posterior segment examination. The AC/A ratio was calculated using the gradient method (+3.00 D lenses).

**Deviation angle measurement:** All deviation angles reported in this study were measured using the prism cover test while participants wore their full cycloplegic hyperopic correction. For patients with refractive AE, this correction fully neutralized the esotropia, resulting in orthotropia (0 prism diopters) at both distance and near. For high AC/A ratio and patients with partially accommodative AE, measurements were obtained under the same corrected conditions to ensure consistency across all subtypes. Distance (6 m) and near (33 cm) deviations were recorded in PD using the alternate prism cover test.

### Classification of AE Subtypes

All deviation measurements were performed with full cycloplegic hyperopic correction in place after 6 to 8 weeks of consistent spectacle wear. Patients were classified according to Awadein et al.: refractive AE (normal alignment with full hyperopic correction and normal AC/A ratio); partially accommodative AE (residual esotropia >10 PD at distance persisting 6–8 weeks after full correction); and high AC/A ratio AE with high AC/A ratio (gradient AC/A >5:1 or near esotropia ≥10 PD greater than distance after full correction).^3^ Binocular fusion was confirmed in all patients with refractive AE using Worth 4-dot testing, and stereopsis was assessed using the Titmus stereo test (Stereo Optical Co., Chicago, IL, USA) where feasible.

### Optical Coherence Tomography Angiography

OCTA imaging was performed using the Topcon DRI OCT-1 Triton swept-source system (1050 nm wavelength, real-time eye-tracking). All scans were performed between 9:00 AM and 12:00 PM by a single experienced technician masked to group status, without pupil dilation. Intra-observer and inter-observer reproducibility were assessed using intraclass correlation coefficients (ICCs) for 20 randomly selected eyes. The mean ICC for intra-observer agreement was 0.94 (range = 0.89–0.97) and for inter-observer agreement was 0.91 (range = 0.85–0.95), indicating excellent reliability.

The protocol captured a 6 × 6 mm macular area centered on the fovea (512 × 256 A-scans). Only images with quality index >40 and no significant motion artifacts or segmentation errors were included. Automated software (IMAGEnet 6, version 1.22) segmented retinal vasculature into four layers: SCP; inner limiting membrane to 15.6 µm below inner plexiform layer (IPL)/inner nuclear layer (INL) junction; DCP; 15.6 to 70.2 µm below this junction; and OR; 70.2 µm below IPL/INL junction to Bruch’s membrane; and CC; Bruch’s membrane to 10.4 µm beneath it.

**Figure 1.**
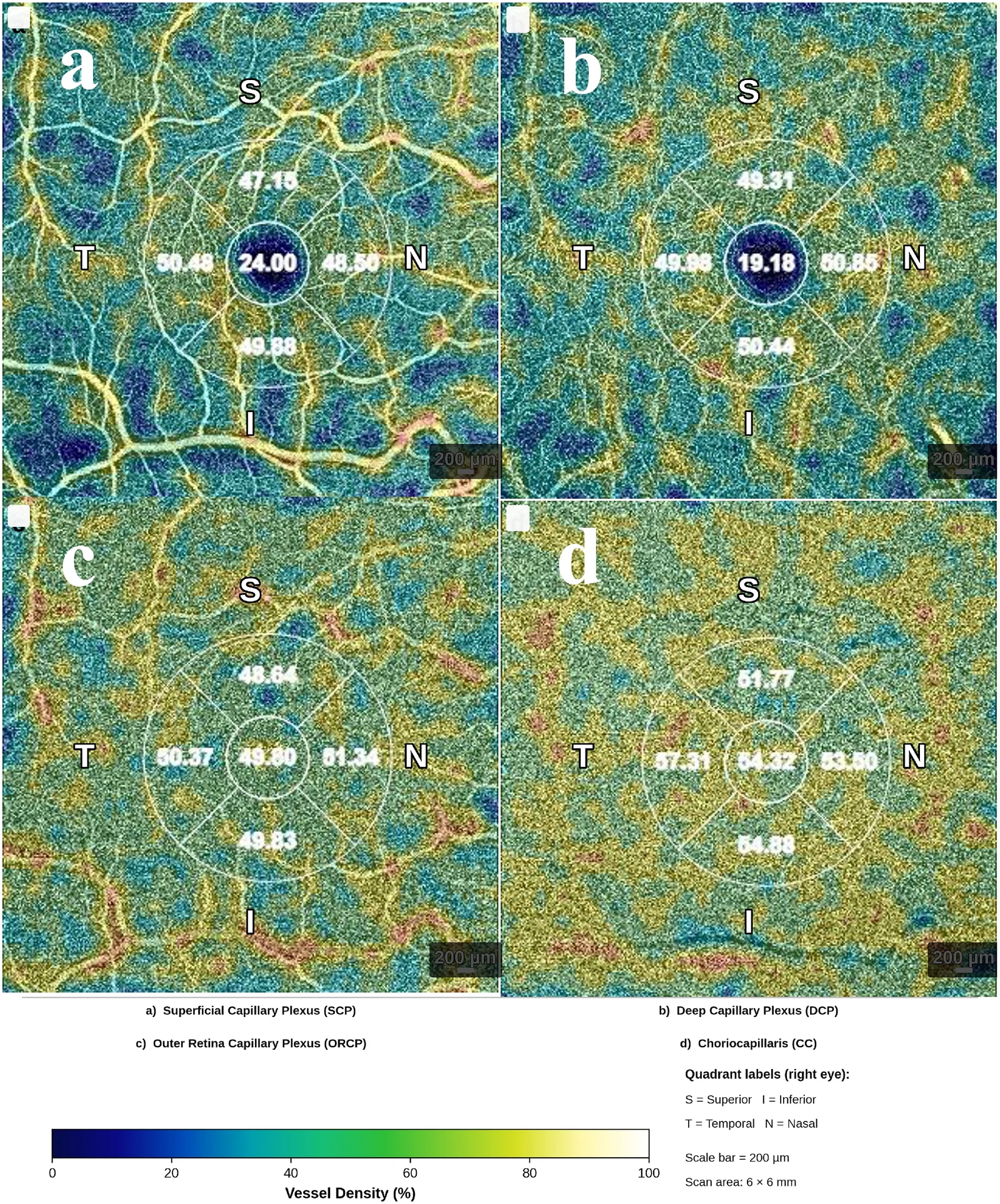
Representative OCTA analysis of a participant with accommodative esotropia in different quadrants (central, nasal, temporal, superior. and inferior; right eye). (**a**) Superficial capillary plexus vessel density. (**b**) Deep capillary plexus vessel density. (**c**) Outer retina capillary plexus vessel density. (**d**) Choriocapillaris vessel density. Scan area = 6 × 6 mm. *Scale bar* = 200 µm.

VD (percentage area occupied by flowing blood vessels) was calculated for each layer across five macular regions using a standardized Early Treatment Diabetic Retinopathy Study (ETDRS) grid: central fovea (1-mm diameter circle), and superior, inferior, nasal, and temporal quadrants (defined by ETDRS division) 3-mm diameter ring within the 6 × 6 mm area. Foveal centration was confirmed by manual alignment of the foveal avascular zone center. All segmentations were reviewed by two independent graders and manually corrected if necessary. The foveal avascular zone (FAZ) area was not analyzed due to software limitations (Fig. 1).

### Statistical Analysis

Analyses were performed using SPSS version 23.0. Normality was assessed with Kolmogorov-Smirnov test. Continuous variables are presented as mean ± SD; categorical variables as frequencies and percentages. Prior to the main analysis, interocular differences between right and left eyes were assessed using paired *t*-tests for all OCTA parameters to confirm the appropriateness of pooling both eyes. Generalized estimating equation (GEE) models with Gaussian distribution, identity link function, and exchangeable working correlation structure were used to account for inter-eye correlations and to adjust for SE refraction and AL as covariates. The Huber-White sandwich estimator was used for robust variance estimation.

### Multiple Comparisons Correction

Given the exploratory nature of this study, we performed 20 separate GEE models (4 retinal layers × 5 macular quadrants). To control for multiple comparisons, we applied the Benjamini-Hochberg false discovery rate (FDR) procedure with q = 0.05 set a priori. Findings with FDR-adjusted q-values < 0.05 were considered statistically significant. A 2-tailed *P* value < 0.05 was used for unadjusted comparisons.

## Results

A total of 34 patients with AE (68 eyes) and 35 age-matched healthy controls (70 eyes) were included after 1 patient with AE was excluded due to poor image quality. The AE subtypes comprised 8 patients with refractive (16 eyes), 10 patients with high AC/A ratio (20 eyes), and 16 patients with partially accommodative (32 eyes). The mean age was comparable between the AE and control groups (9.2 ± 2.3 years vs. 9.8 ± 2.8 years, respectively; *P* = 0.356). The AE group exhibited a significantly more hyperopic SE refraction compared with controls (3.1 ± 1.6 D vs. −0.62 ± 1.1 D; *P* < 0.0001). AL measurements were 21.9 ± 0.8 mm in the AE group and 23.2 ± 1.1 mm in the control group, a difference that was statistically significant (*P* < 0.0001). Right and left eyes were compared for all OCTA parameters across four layers and five quadrants. No significant interocular differences were found for any parameter in the overall AE group or within any subtype (all *P* > 0.05, paired *t*-test), supporting the use of GEE models to account for inter-eye correlation. In the refractive AE subgroup (*n* = 8), all patients were orthotropic (0 PD) with full correction at OCTA imaging. Six of eight patients had measurable stereopsis (median = 60 arcsec, range = 40–100 arcsec), and 2 had gross stereopsis (400 arcsec) with stable Worth 4-dot fusion. No patient had diplopia or manifest deviation with correction. Demographic and clinical characteristics are summarized in Table 1.

**Table 1.** Demographic and Clinical Characteristics of Study Groups

Parameter	AE Group, *n* = 34	Refractive AE, *n* = 8	High AC/A Ratio AE, *n* = 10	Partially Accom. AE, *n* = 16	Control Group, *n* = 35	*P* Value, AE Vs. Control^*^	*P* Value, Among Subtypes^†^
Age, y	9.2 ± 2.3	8.9 ± 2.1	9.4 ± 2.5	9.1 ± 2.4	9.8 ± 2.8	0.356	0.742
Sex, M/F	18/16	4/4	5/5	9/7	18/17	0.892	0.915
SE, D	3.1 ± 1.6	3.2 ± 1.5	3.0 ± 1.7	3.1 ± 1.6	−0.62 ± 1.1	**<0.0001**	0.892
AL, mm	21.9 ± 0.8	22.0 ± 0.7	21.8 ± 0.9	21.9 ± 0.8	23.2 ± 1.1	**<0.0001**	0.856
BCVA, logMAR	0.02 ± 0.04	0.02 ± 0.03	0.03 ± 0.05	0.02 ± 0.04	0.01 ± 0.03	0.234	0.687
Deviation without correction, PD, distance	—	23.4 ± 6.2	28.1 ± 5.4	30.2 ± 6.1	—	—	0.089
Deviation without correction, PD, near	—	25.1 ± 7.3	38.2 ± 5.9	33.6 ± 6.8	—	—	**0.003**
Deviation angle, PD, distance	18.4 ± 6.2	0	22.3 ± 4.8	24.1 ± 5.6	—	—	**<0.0001**
Deviation angle, PD, near	24.7 ± 8.1	0	32.5 ± 6.3	28.4 ± 5.9	—	—	**<0.0001**
AC/A ratio	4.8 ± 1.6	3.2 ± 0.8	6.4 ± 1.2	4.1 ± 1.1	—	—	**<0.0001**

AL, axial length; BCVA, best-corrected visual acuity; PD, prism diopter; SE, spherical equivalent.

Values are presented as mean ± SD. Deviation angles were measured with full cycloplegic hyperopic correction in place. Deviation angles without correction represent baseline measurements at initial presentation. The 0 PD in refractive AE indicates orthotropia with full correction at OCTA imaging. Stereopsis was confirmed in all eight patients (median = 60 arcsec, range = 40–400 arcsec).

Bold indicates statistical significance (*P* < 0.05).

* *P* value represents comparison between overall AE group and control group (GEE models).

† *P* value among the three AE subtypes only with no control reference (GEE models).

**Table 2A.** OCTA Vessel Density Measurements (%). Overall AE vs. Control Group

Layer	Quadrant	AE Overall, *n* = 34	Control, *n* = 35	Unadjusted *P* Value	Adjusted *P* Value^*^
SCP	Central	21.6 ± 3.9	22.0 ± 5.2	0.674	0.673
	Nasal	46.1 ± 3.7	45.9 ± 3.2	0.726	0.726
	Temporal	47.5 ± 3.3	47.6 ± 2.8	0.849	0.850
	Superior	45.6 ± 3.1	46.4 ± 3.7	0.166	0.165
	Inferior	45.2 ± 4.2	45.2 ± 3.3	0.932	0.931
DCP	Central	15.2 ± 4.8	16.6 ± 5.2	0.168	0.166
	Nasal	48.2 ± 3.9	48.6 ± 3.2	0.547	0.545
	Temporal	48.3 ± 4.5	48.1 ± 3.4	0.790	0.795
	Superior	47.1 ± 3.7	48.2 ± 3.4	0.063	0.715
	Inferior	47.3 ± 4.3	48.3 ± 2.6	0.132	0.134
OR	Central	46.5 ± 3.4	47.4 ± 2.9	0.169	0.170
	Nasal	50.8 ± 2.2	51.1 ± 1.9	0.501	0.543
	Temporal	50.9 ± 2.9	51.1 ± 1.8	0.866	0.892
	Superior	**49.4 ± 1.6**	**50.2 ± 2.0**	**0.029**	**0.239**
	Inferior	49.9 ± 2.1	50.2 ± 1.7	0.346	0.387
CC	Central	**53.7 ± 3.3**	**54.7 ± 2.4**	**0.036**	**0.986**
	Nasal	53.7 ± 1.9	54.2 ± 1.9	0.167	0.204
	Temporal	54.1 ± 2.6	54.3 ± 2.1	0.631	0.672
	Superior	**51.2 ± 2.3**	**52.8 ± 2.5**	**<0.0001**	**0.001**
	Inferior	53.1 ± 2.9	53.2 ± 1.7	0.8	0.912

CC, choriocapillaris; DCP, deep capillary plexus; OR, outer retina; SCP, superficial capillary plexus.

Values are presented as mean ± SD.

Bold indicates statistically significant findings (*P* < 0.05).

* Adjusted *p* value: GEE adjustment for spherical equivalent and axial length.

**Table 2B.** OCTA Vessel Density Measurements (%). AE Subtypes vs. Control Group (Superior Quadrant Only)

Layer	Refractive AE, *n* = 8	High AC/A Ratio AE, *n* = 10	Partially Accom. AE, *n* = 16	Control, *n* = 35	*P* Value^*^	*P* Value Ref^†^	*P* Value High AC/A Ratio^†^	*P* Value Part^†^
SCP superior	45.8 ± 2.8	45.9 ± 2.9	45.3 ± 3.4	46.4 ± 3.7	0.863	0.892	0.845	0.917
DCP superior	48.4 ± 4.1	48.2 ± 3.1	**45.6 ± 3.4**	48.2 ± 3.4	**0.006**	0.627	0.842	**0.027**
CC superior	**51.2 ± 3.1**	**51.5 ± 1.7**	**50.9 ± 2.2**	52.8 ± 2.5	**0.005**	**0.041**	**0.005**	**<0.0001**
OR superior	50.3 ± 2.1	49.2 ± 1.8	50.5 ± 2.3	50.2 ± 2.0	**0.010**	0.167	**0.010**	0.631

Values are presented as mean ± SD.

Bold indicates statistically significant findings (*P* < 0.05).

These are uncorrected *P* values. For FDR-corrected q-values, see Table 2d.

* *P* values overall comparison among four groups (Kruskal-Wallis test).

† *P* values ref, *P* values high AC/A ratio, *P* values part: comparison of each subtype versus control group (GEE adjusted for SE and AL).

**Table 2C.** OCTA Vessel Density Measurements (%). Summary of Other Quadrants

Layer	Quadrant	Refractive AE	High AC/A Ratio AE	Partially Accom. AE	Control	*P* Value
All layers	Central	>0.05	>0.05	>0.05	Reference	>0.05
	Nasal	>0.05	>0.05	>0.05	Reference	>0.05
	Temporal	>0.05	>0.05	>0.05	Reference	>0.05
	Inferior	>0.05	>0.05	>0.05	Reference	>0.05

All layers *P* > 0.05.

No statistically significant differences were found in any other quadrants or layers across all groups (all *P* > 0.05).

### Comparisons Between AE and Control Eyes

In the SCP, VD measurements were comparable between the AE and control groups across all quadrants (central, *P* = 0.674, nasal, *P* = 0.726, temporal, *P* = 0.849, superior, *P* = 0.166, and inferior, *P* = 0.932; all *P* > 0.05). Similarly, no significant intergroup differences were observed in the DCP for any region (central, *P* = 0.168, nasal, *P* = 0.547, temporal, *P* = 0.790, superior, *P* = 0.063, and inferior, *P* = 0.132; all *P* > 0.05).

Analysis of the OR layer revealed a significantly reduced VD in the AE group compared with controls, but this was confined to the superior quadrant (*P* = 0.029). No significant differences were found in the central (*P* = 0.169), nasal (*P* = 0.501), temporal (*P* = 0.866), or inferior (*P* = 0.346) quadrants. However, in GEE models adjusting for SE and AL, this superior quadrant reduction did not retain statistical significance (*P* = 0.239).

**Figure 2.**
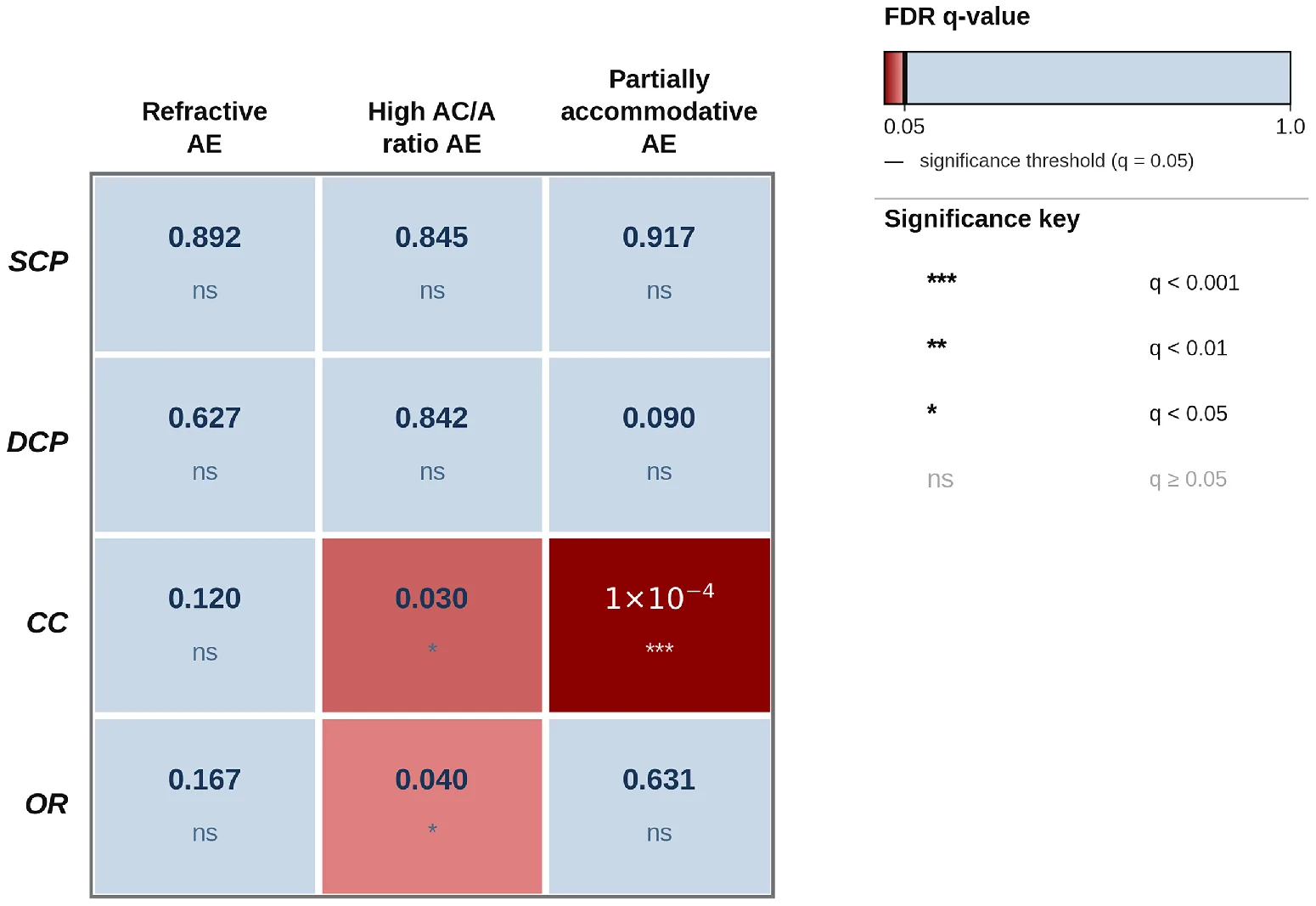
Macular microvascular alterations in the superior quadrant across accommodative esotropia (AE) subtypes. Heatmap displays FDR-corrected q-values derived from comparisons between patients and controls for each retinal layer group: superficial capillary plexus (SCP), deep capillary plexus (DCP), choriocapillaris (CC), and outer retina (OR). *Columns* represent AE subtypes: refractive AE, high AC/A ratio AE, and partially accommodative AE. Color intensity encodes significance level; *red* indicates q < 0.05 (statistically significant). *Asterisks* denote significance thresholds after FDR correction (Benjamini–Hochberg method). AE, accommodative esotropia; AC/A, accommodative convergence/accommodation ratio; FDR, false discovery rate.

In the CC layer, VD was generally similar among groups. Whereas the nasal (*P* = 0.167), temporal (*P* = 0.631), and inferior (*P* = 0.8) quadrants showed no significant differences, the AE group exhibited significantly lower VD in the central (*P* = 0.036) and superior (*P* < 0.0001) quadrants. After adjusting for SE and AL, the reduction in the superior quadrant remained significant (*P* = 0.001), whereas the central quadrant finding did not (*P* = 0.986). These results are illustrated in Figure 2 and summarized in Table 2.^2^,^3^,^4^

### Comparisons Between AE Subtypes and Control Eyes

The following subgroup analyses were exploratory and not powered for definitive conclusions. Findings should be interpreted cautiously. In the SCP, no significant differences in VD were found among any of the three AE subtypes and the control group across all quadrants (all *P* > 0.05). For the DCP, significant intergroup differences were confined to the superior quadrant (*P* = 0.006). After adjusting for SE and AL and applying Benjamini-Hochberg FDR correction (q = 0.05), this finding did not retain statistical significance (mean difference = −2.6%, 95% confidence interval [CI] = −4.9% to −0.3%, Cohen’s d = 0.71, *P* = 0.027, q = 0.09). No significant differences were observed in the other quadrants (all *P* > 0.05). In the OR layer, significant intergroup differences were confined to the superior quadrant (*P* = 0.010). After FDR correction, this finding remained statistically significant (mean difference = −1.0%, 95% CI = −1.8% to −0.2%, Cohen’s d = 0.55, *P* = 0.010, q = 0.04). No significant differences were observed in the other quadrants (all *P* > 0.05).

Evaluation of the CC layer revealed a significantly reduced VD, limited to the superior quadrant, in all three AE subtypes compared with controls (*P* = 0.005).

After FDR correction, this reduction remained significant for the high AC/A ratio (mean difference = −1.3%, 95% CI = −2.2% to −0.4%, Cohen’s d = 0.61, *P* = 0.005, q = 0.03) and partially accommodative (mean difference = −1.9%, 95% CI = −2.7% to −1.1%, Cohen’s d = 0.85, *P* < 0.0001, q < 0.001) subtypes, but not for the refractive subtype (mean difference = −1.6%, 95% CI = −3.1% to −0.1%, Cohen’s d = 0.52, *P* = 0.041, q = 0.12). Results are summarized in Tables 2a–d. Correlation analyses revealed no significant correlations between superior quadrant CC or OR VD and deviation angle at distance (*r* = −0.12 to −0.09, *P* > 0.05), deviation angle at near (*r* = −0.19 to −0.15, *P* > 0.05), or AC/A ratio (*r* = −0.18 to −0.07, *P* > 0.05).

**Table 2D.** OCTA Vessel Density Measurements (%). Significant Findings With Benjamini-Hochberg FDR Correction (Superior Quadrant Only)

Comparison	Layer	Mean Difference, AE - Control	95% CI	Cohen.s d	Uncorrected *P* Value	FDR q-Value^*^
Refractive AE vs. control	CC	−1.6%	−3.1% to −0.1%	0.52	0.041	0.12
High AC/A ratio AE vs. control	CC	−1.3%	−2.2% to −0.4%	0.61	0.005	**0.03**
High AC/A ratio AE vs. control	OR	−1.0%	−1.8% to −0.2%	0.55	0.010	**0.04**
Partially accom. AE vs. control	DCP	−2.6%	−4.9% to −0.3%	0.71	0.027	0.09
Partially accom. AE vs. control	CC	−1.9%	−2.7% to −1.1%	0.85	<0.0001	**<0.001**

CC, choriocapillaris; DCP, deep capillary plexus; OR, outer retina.

Bold indicates statistical significance (q < 0.05).

* Benjamini-Hochberg false discovery rate (FDR) correction applied across 20 separate GEE models (4 layers × 5 quadrants) with q = 0.05 set a priori.

## Discussion

This study provides the first evaluation of macular microvascular alterations in children with AE using OCTA. Our key findings reveal layer-specific and quadrant-specific vascular changes. After applying Benjamini-Hochberg FDR correction (q = 0.05) to control for multiple comparisons, significant VD reductions were observed in the CC across the high AC/A ratio (*P* = 0.005, q = 0.03) and partially accommodative (*P* < 0.0001, q < 0.001) subtypes, confined to the superior quadrant. The OR reduction in the high AC/A ratio subtype also remained significant (*P* = 0.010, q = 0.04). The CC reduction in the refractive subtype (*P* = 0.041, q = 0.12) and the DCP reduction in the partially accommodative subtype (*P* = 0.027, q = 0.09) did not survive FDR correction. These findings suggest distinct, subtype-specific microvascular alterations independent of refractive and biometric confounders, though these subgroup findings require confirmation in larger, adequately powered studies.

The observed VD reductions were relatively small (1%–2% absolute difference). Whereas statistically significant after FDR correction for CC and OR findings, the clinical relevance of these small changes remains uncertain. Whether such differences translate into clinically meaningful functional impairment or predict treatment response requires further investigation in larger prospective studies.

The predilection for superior quadrant involvement across multiple retinal layers is noteworthy. Although no prior OCTA studies in AE exist for direct comparison, our findings align with emerging evidence of structural ocular changes in strabismic populations.^6^^,^^7^ Wen and colleagues demonstrated increased thickness of nasal intraretinal layers in concomitant esotropia, suggesting region-specific retinal alterations.^7^ Al-Haddad and colleagues reported smaller optic nerve head cup volume and cup-to-disc ratio in esotropic children independent of refraction and AL—a pattern analogous to our microvascular reductions that withstood confounder adjustment.^6^ The superior quadrant specificity may relate to biomechanical effects of sustained convergence, as Lim and colleagues demonstrated significant optic disc and vascular strain during horizontal duction that could influence regional perfusion.^12^ Potential artifacts (scan decentration, fixation instability, eyelid interference, motion artifacts, segmentation errors, and multiple comparisons) cannot be entirely excluded. However, our quality control procedures and the layer-specific, subtype-specific patterns suggest a biological origin, particularly as the CC findings survived FDR correction.

The differential layer involvement across AE subtypes provides insights into heterogeneous pathophysiology. CC reductions across the high AC/A ratio and partially accommodative subtypes suggest CC changes may represent a key finding in AE, particularly in more severe or established cases. The OR layer specificity for high AC/A ratio (high AC/A ratio) cases, which survived FDR correction (*P* = 0.010, q = 0.04), may relate to distinct neural mechanisms underlying convergence excess.^3^ In contrast, the DCP finding in partially accommodative AE (*P* = 0.027, q = 0.09) did not survive correction and is therefore considered hypothesis-generating. These findings complement Koc and colleagues, who demonstrated shallower anterior chambers and limited lens forward movement during accommodation in refractive AE, suggesting structural limitations accompany the microvascular alterations we observed.^4^ The absence of significant correlations between OCTA parameters and clinical measures may reflect the modest sample size within each subtype or suggest that microvascular changes represent an independent biological phenomenon not directly related to deviation magnitude and AC/A ratio.

The relationship between amblyopia and retinal microvasculature warrants consideration. Simsek and colleagues reported reduced SCP and DCP VD in various amblyopia types with negative correlations between visual acuity and VD.^10^ Our study excluded amblyopia to isolate AE effects, yet still found significant alterations, suggesting strabismus independent of amblyopia influences retinal perfusion. However, our finding that reductions were confined to deeper layers rather than SCP—which was prominently affected in Simsek’s amblyopic cohort—suggests amblyopia and strabismus may differentially impact retinal microvasculature.^10^ This distinction is supported by Niyaz and colleagues, who reported thicker choroids in anisometropic amblyopic eyes but not in strabismic amblyopia, indicating that choroidal changes accompanying strabismus differ fundamentally from those in anisometropia.^13^

The longitudinal work of Wang and colleagues demonstrating that ocular biometric components develop at different rates in infantile versus late-onset AE provides context for our subtype-specific findings.^5^ Slower axial growth in late-onset AE may be accompanied by different microvascular development patterns, potentially explaining distinct vascular signatures across subtypes.

This study has several notable strengths. First, it represents the first comprehensive evaluation of macular microvascular alterations in children with AE using OCTA, addressing a significant gap in the literature. Additional strengths include its prospective design, a priori sample size calculation, strict exclusion of amblyopia, and confounding pathologies, GEE models accounting for inter-eye correlations while adjusting for refractive error and AL, classification into three AE subtypes enabling detection of differential vascular patterns.

Limitations include cross-sectional design precluding assessment of temporal relationships. Due to the cross-sectional design, causality cannot be inferred from the observed associations*.* Modest sample size within each subtype potentially limits detection of subtle variations. Despite statistical adjustment for SE and AL, residual confounding due to refractive and AL differences may persist, as OCTA measurements remain sensitive to magnification and segmentation effects related to ocular biometry. Prism fusion ranges were not systematically recorded for all participants, and stereoacuity data were incomplete in some patients, limiting the assessment of binocular function across all subgroups. Correlations with stereoacuity and Worth 4-dot were not performed as these data were not available for all participants. Inability to analyze the FAZ area due to software limitations, age range encompassing developmental variation, and absence of functional correlation with binocular vision parameters limit links between structural findings and visual function. An additional limitation is the modest sample size within each AE subtype (refractive AE, *n* = 8; high AC/A ratio AE, *n* = 10; and partially accommodative AE, *n* = 16), which provided limited statistical power for detecting moderate effect sizes in subgroup comparisons. Post hoc power analysis indicated that for the smallest subgroup (refractive AE, *n* = 8), the detectable effect size (Cohen’s d) was approximately 1.2 for two-group comparisons with α = 0.05 and power = 0.80. Therefore, the absence of significant findings in some subgroup comparisons should not be interpreted as evidence of no effect, and the significant findings reported require replication in larger cohorts. Fixation patterns (consistent deviating versus alternating fixation) were not systematically recorded. However, interocular equivalence of OCTA parameters was confirmed statistically (all *P* > 0.05), and visual acuity and refractive errors were symmetrical between eyes.

The single-center design may also limit generalizability to broader populations with different demographic or clinical characteristics. Detailed data on duration of spectacle wear and quantitative compliance measures were not collected, which limits the assessment of long-term refractive management effects on microvascular outcomes. Furthermore, we excluded children with hyperopia exceeding +6.00 D SE to avoid confounding by high refractive error and to ensure reliable OCTA signal quality. This exclusion criterion introduces a selection bias, as some patients with fully refractive AE—who often present with moderate to high hyperopia—were not represented. Therefore, our findings may not generalize to patients with AE with very high hyperopic refractive errors (>+6.00 D).

### Clinical Implications

The consistent involvement of the CC across high AC/A ratio and partially accommodative AE subtypes—which remained significant after FDR correction—raises the possibility that CC VD measured by OCTA could serve as a biomarker of chronic accommodative effort. If validated in longitudinal studies, this parameter might help quantify disease burden or monitor treatment response. Additionally, the superior quadrant predominance suggests that clinicians interpreting OCTA in patients with AE should focus attention on this region rather than the fovea alone.

## Conclusions

Children with AE show significant, layer-specific microvascular alterations predominantly confined to the superior quadrant, with distinct patterns across AE subtypes independent of refractive and biometric confounders. Although these exploratory observations raise intriguing possibilities regarding heterogeneous pathophysiology, they should be considered hypothesis-generating pending validation in larger cohorts. These findings extend evidence that strabismus is associated with structural ocular changes beyond extraocular muscles, encompassing retinal microvasculature. Future longitudinal studies should investigate reversibility of these alterations with successful treatment and correlation with binocular functional outcomes.
